# An intelligent guided troubleshooting method for aircraft based on HybirdRAG

**DOI:** 10.1038/s41598-025-02643-2

**Published:** 2025-05-22

**Authors:** Xiaoyue Xie, Xilang Tang, Siwei Gu, Lijie Cui

**Affiliations:** 1 School of Equipment Management and UAV Engineering, Air Force Engineering University, Xi’an, 710051 China; 2 Hangzhou Joyful Data Technology Corporation, Hangzhou, 310018 China

**Keywords:** Knowledge graph, HybridRAG, Multi-Dimensional retrieval, Guided troubleshooting, Aerospace engineering, Computer science, Information technology

## Abstract

To enhance aircraft fault diagnosis efficiency, this paper proposes HybridRAG, an intelligent-guided troubleshooting framework that integrates knowledge graphs and large language models (LLMs). Unlike conventional retrieval-augmented generation (RAG) methods that rely on single-modal retrieval, HybridRAG adopts a multi-dimensional retrieval strategy, combining graph-based reasoning with both vector-based and BM25-based text retrieval techniques. This hybrid approach ensures comprehensive extraction of relevant information from both unstructured text and structured fault graphs, enhancing diagnostic precision, relevance, and robustness. Experimental results demonstrate that HybridRAG achieves an F1 score improvement of at least 4% and reduces hallucination rates by over 7% compared to mainstream RAG baselines. These advancements, combined with its unique integration of multi-modal retrieval, position HybridRAG as a novel framework for addressing complex aircraft maintenance challenges. Additionally, the paper presents an agent-based intelligent troubleshooting assistant that supports more interactive, adaptive, and flexible diagnostic Q&A, providing maintenance personnel with a significant advanced intelligent, context-aware diagnostic tool.

**Supplementary Information:**

The online version contains supplementary material available at 10.1038/s41598-025-02643-2.

## Introduction

As aircraft systems grow increasingly complex, fault diagnosis has become a typical knowledge-intensive activity. When facing unknown faults, maintenance personnel often spend considerable time analyzing technical documents pertaining to these anomalies. These documents provide essential details about system component composition, signal parameter variations, and historical fault case records. This time-consuming process leads to extended equipment downtime, significantly affecting aircraft availability and operational efficiency. Consequently, enhancing the efficiency and accuracy of fault diagnosis is crucial for ensuring aircraft stability and reliability.

To address these challenges, various intelligent diagnostic methods have been developed to assist maintenance personnel in more efficient fault identification and resolution. Early approaches primarily relied on rule-based system (RBS) and case-based reasoning (CBR). RBS achieves efficient diagnosis through predefined logic trees, proving particularly effective for routine fault scenarios^1^. However, its rigid knowledge representation imposes limitations when handling novel complex faults. In contrast, CBR leverages historical data similarity matching mechanisms, demonstrating stronger adaptability in unknown fault diagnosis^2^, though its diagnostic accuracy remains highly dependent on the quality and comprehensiveness of the case database. To synergize the strengths of both approaches, hybrid expert systems emerged, integrating rule-based reasoning with case-based reasoning^3^. This combination improved diagnostic accuracy and adaptability, but it struggled to cope with the escalating complexity of aircraft systems and the ever-expanding of technical knowledge required.

The emergence of knowledge graph (KG) technology has driven extensive research into domain-specific expert system construction^4–7^. By establishing structured KGs paired with advanced reasoning algorithms, these systems help maintenance personnel reduce time spent on fault information retrieval, accelerate fault localization rapidly localizing faults, and enhance diagnostic efficiency during equipment operations. However, while knowledge graph-based systems streamline information retrieval and partially boost productivity, they ultimately remain constrained as static retrieval tools^8–10^, lacking flexible natural multi-turn dialogue mechanisms and unable to provide step-by-step guidance for complex fault isolation. For instance, when diagnosing abnormal brake pressure, traditional KG approaches execute static entity resolution—mapping symptoms to related entities such as brake assemblies, hydraulic sensors, and control valves—followed by template-driven slot filling to retrieve relevant fault cases. This retrieval paradigm, relying on keyword matching or fixed vector similarity metrics, fails to incorporate dynamic contextual cues or adapt reasoning paths through interactive dialogue. These limitations motivate the integration of large language models (LLMs), which offer flexible reasoning capabilities and natural multi-turn conversations for guided troubleshooting. Despite LLMs’ well-documented success in general NLP tasks^11–14^, their direct deployment for aircraft maintenance diagnostics faces significant barriers. Primary among these are inherent domain knowledge deficiencies — particularly regarding aircraft-specific fault patterns — that may compromise diagnostic reliability^15^, coupled with the intrinsic opacity of neural network decision-making processes that undermines traceability in safety-critical scenarios^16^. In comparison, domain-specific KGs (e.g., AFKG^7^, AAKG^17^) provide structured knowledge representation via graph databases with transparent reasoning processes. This complementary relationship—combing KG’s structured expertise with LLM’s adaptive reasoning—creates a promising framework for aircraft fault diagnosis, enabling maintenance personnel to leverage both technologies for substantial improvements in troubleshooting accuracy and operational efficiency.

Current methodologies for enhancing LLMs with KGs fall into two paradigms: knowledge infusion and retrieval-augmented generation (RAG). The knowledge infusion approach embeds KG elements directly into LLM pretraining to enhance domain knowledge retention, but faces critical constraints including the massive parameter sizes of modern LLMs requiring prohibitive computational resources for iterative updates, and the inherent data disparity between compact domain-specific KGs and LLMs’ pretraining corpora that mandates meticulous prompt engineering to activate latent knowledge^18^. In contrast, RAG dynamically fetches relevant KG subgraphs during inference, supplementing LLMs with contextual knowledge^19^, and demonstrates several advantages on real-time knowledge integration synchronization, cost-effective deployment, and auditable decision trails through explicit retrieval evidence^20,21^. These benefits make RAG particularly suited for aviation maintenance’s dynamic environments. Given the cost-effectiveness and need for transparency in fault diagnosis, we prioritize RAG over KG embedding for our approach. Although a hybrid framework combining KG embedding and retrieval-based augmentation, as proposed in^22^, appears promising for integrating structured knowledge into large models, it has notable drawbacks. Its effectiveness hinges on high-quality domain-specific KGs, which require continuous updates. Adapting the LLM to evolving knowledge still demands parameter adjustments, incurring significant computational costs. Moreover, unlike aviation assembly, our focus is on real-time fault diagnosis, where users need interactive, step-by-step guidance. This process relies on LLMs’ general reasoning and conversational abilities, which can be hindered by subgraph embedding, limiting flexibility and adaptability in troubleshooting. Therefore, we aim to enhance fault diagnosis accuracy and streamline troubleshooting guidance by designing an advanced retrieval-augmented strategy while preserving the reasoning capabilities of the underlying large language model.

This study presents HybridRAG, an intelligent guided troubleshooting framework for aircraft maintenance that achieves an optimal balance between diagnostic accuracy with transparency. The framework establishes a domain-specific fault knowledge graph and implements a novel multi-dimensional retrieval strategy combining vector, BM25 text, and graph retrieval techniques. This hybrid approach comprehensively extracts and synthesizes relevant information from both unstructured text and structured graph data, enabling large language models to deliver more precise and context-aware fault diagnosis. Experimental results demonstrate that our hybrid retrieval strategy, outperforms several mainstream RAG methods in both diagnostic accuracy and interpretability. Furthermore, we develop an agent-based intelligent troubleshooting assistant within this framework, incorporating advanced interactive capabilities to provide adaptive, step-by-step fault localization guidance. Practical evaluations across multiple scenarios, especially in complex fault situations. In summary, this paper primarily contributes in three main areas.

- Addressing efficiency and accuracy issues in aircraft fault diagnosis: The paper addresses challenges encountered by traditional knowledge-based expert systems in complex diagnostic scenarios, such as rigid interaction methods and a lack of multi-round dialogue capabilities. It also considers potential accuracy and authenticity issues that may arise when applying large language models directly.
- Proposing a HybirdRAG-based intelligent guided troubleshooting framework: This framework merges the creation of domain-specific fault knowledge graphs with multi-dimensional retrieval strategies, including vector, BM25 text, and a novel graph exploration retrieval algorithm and highly optimized algorithm implementation.
- Significant advantages demonstrated in comparative experiments: Experimental results demonstrate that HybirdRAG achieves superior performance compared to mainstream RAG baselines in terms of fault diagnosis accuracy and hallucination. Moreover, particularly outstanding effectiveness in complex fault scenarios through our developed agent-based intelligent assistant, which provides adaptive, context-aware troubleshooting guidance.

## Related work

RAG techniques have gained widespread adoption across multiple domains where output reliability is critical, including clinical decision support^23^, financial risk assessment^24^, and personalized education^25^. In aviation maintenance, RAG-based systems have demonstrated potential in accelerating fault diagnosis through the integration of historical case databases with technical documentation. However, their application in safety-critical aviation environments remains challenged by the need for more sophisticated retrieval architectures.

Conventional RAG implementations, such as VectorRAG and BM25-based RAG, primarily employ dense vector similarity search or keyword matching for knowledge retrieval. These methods excel in terms of efficiency and semantic/keyword alignment capabilities. For instance, VectorRAG can identify semantically similar historical cases (e.g., “engine temperature anomaly”) within seconds, while BM25 effectively retrieves precise sections from maintenance manuals based on specific fault codes (e.g., “ECAM code 501”). However, their principal limitation lies in the inability to explicitly represent causal or structural dependencies within knowledge bases^26^, often yielding disconnected troubleshooting suggestions. For instance, while VectorRAG might concurrently retrieve fragments related to “fuel pump failure” and “ECAM warning code 501”, it cannot generate a causal path like “fuel pump failure → insufficient fuel pressure→ECAM 501 trigger”. Similarly, BM25, being heavily reliant on keyword matching, struggles to produce engineering-logical troubleshooting workflows (e.g., “check wear-prone components first, then investigate system faults”). Although recent enhancements incorporate metadata filtering or hybrid search algorithms^27,28^, these still underperform with heterogeneous aviation corpora, frequently producing inaccurate or incomplete diagnosis analyses.

GraphRAG has consequently emerged as a promising alternative, leveraging structured knowledge graphs to model fault causality for aviation maintenance. The current state-of-the-art in graph retrieval predominantly adopts a subgraph extraction approach, which entity linking first identifies query-relevant nodes followed by local subgraph expansion^29,30^. Although effective for capturing localized knowledge, this paradigm frequently fails to preserve long-range dependencies^31–33^. This limitation has spurred reasoning-enhanced graph methods like ROG^34^, TOG^35^ and COE^36^, which combine LLMs with KGs to perform multi-hop inference and path planning—capabilities particularly valuable for tracing indirect fault relationships.

Nevertheless, the efficacy of graph-based retrieval remains fundamentally constrained by the knowledge density within the graph structure. Sparse knowledge representations, exemplified by the fault graph in^7^, where “test method” entities contain only abstract attributes (“operations” or “judgments”) without operational specifics (e.g., detailed workflows or procedural examples), often necessitate recourse to original unstructured text data. This underscores the critical need to augment graph retrieval with document retrieval for comprehensive, step-by-step troubleshooting support. Moreover, certain knowledge sources, such as conversational data like operator-expert troubleshooting dialogues, are not well-suited for direct knowledge graph extraction. The resource-intensive nature of KG construction further compounds these limitations, rendering exhaustive knowledge extraction from all documents impractical. Notably, since knowledge updates predominantly occur in textual formats, direct retrieval from unstructured texts frequently proves more efficient than perpetual graph reconstruction. These considerations collectively demonstrate the complementary advantages of integrating knowledge graph and document retrieval for fault diagnosis applications. Consequently, our work proposes a novel multi-dimensional retrieval paradigm within the RAG framework that synergistically combines vector-based, BM25, and graph-based approaches. This integrated strategy is specifically designed to deliver more robust and efficient troubleshooting support by capitalizing on the respective strengths of each retrieval modality.

### Method

**Fig. 1:**
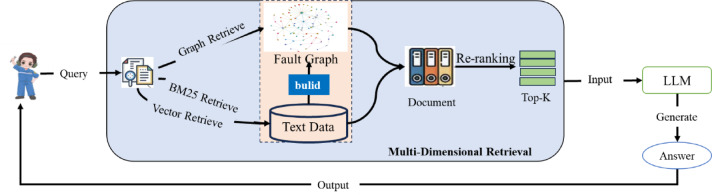
HybirdRAG-based Troubleshooting Framework for Aircraft.

As illustrated in Fig. 1., the HybridRAG framework for aircraft guided troubleshooting comprises three core stages: domain knowledge graph construction, multi-dimensional retrieval (graph retrieval, vector retrieval, and BM25 retrieval), and answer generation. The entire process involves that it conducts multi-dimensional retrieval based on the query of user and place the Top-K retrieval results into prompting instructions of the large language model (LLM), which then generates an answer based on this foundation and returns it to the user.

### *A. Construction of Equipment Fault Graph*

1) Graph scheme development.

**Fig. 2:**
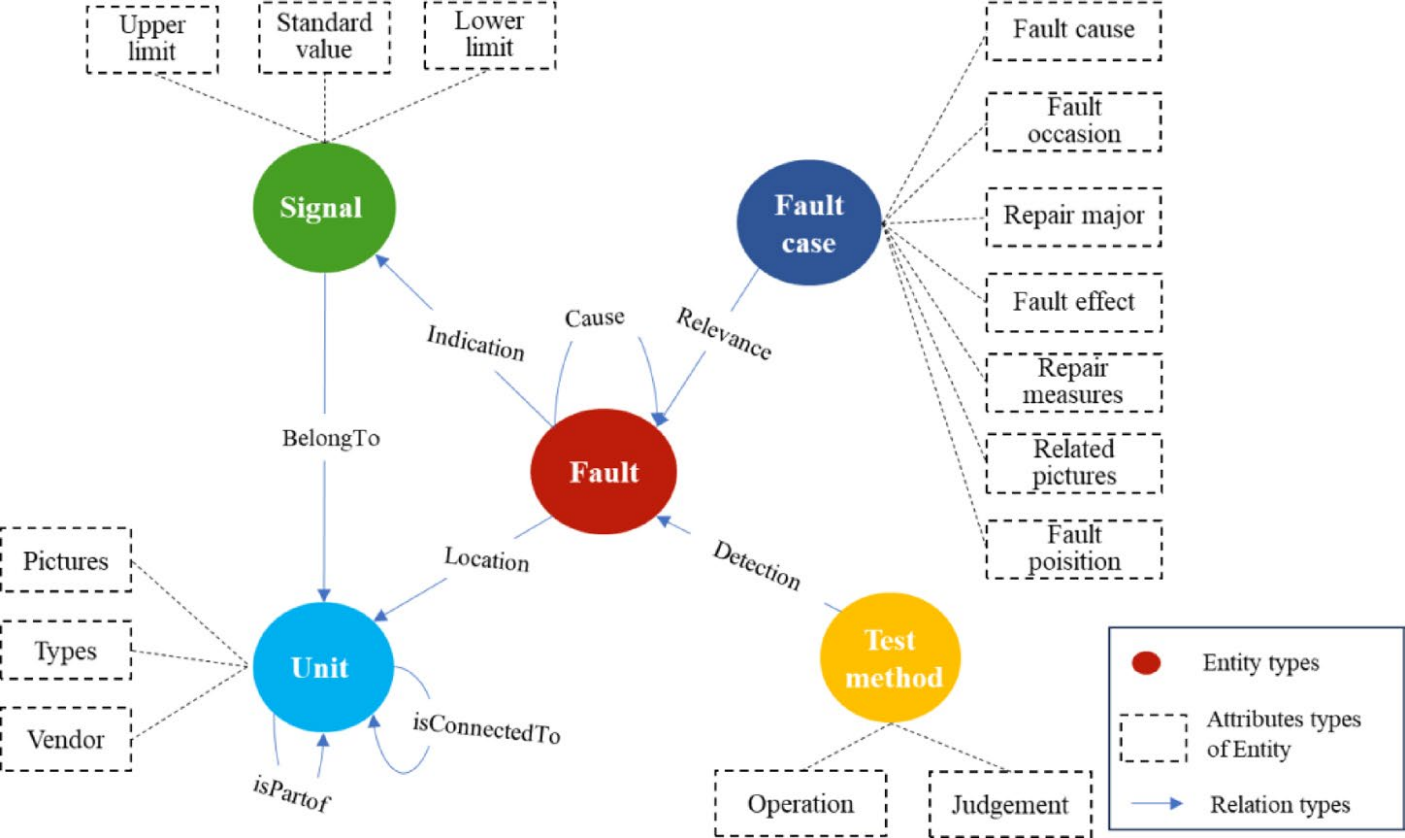
The schema of fault knowledge graph. This schema consists of three types—entities, relationships, and entity attributes—where blue circles represent entity types, orange squares represent entity attributes, and blue arrows represent the relationship types between entities.

To construct an effective fault knowledge graph, we systematically collected and organized aircraft technical documentation from manufacturers, maintenance facilities, regulatory agencies, and academic institutions. These materials encompass equipment configurations, operational principles, historical fault cases, and maintenance protocols, forming a comprehensive knowledge base that ensures both practical utility and broad applicability. Following the methodology outlined in^7^, we develop an aircraft fault ontology model comprising 5 entity types, 8 relationship categories, and 18 attribute classes across structural, functional, and fault dimensions (Fig. 2). Detailed specifications of entities, relationships, and attributes are provided in Appendix A.

### 2) Entity-Relation Triple Extraction

Entity-relation triple extraction identifies entity-relation-entity structures (e.g., < Fault, Location, Unit>) from unstructured text, serving as the critical process in knowledge graph construction. While deep neural networks excel at this task through advanced semantic encoding, conventional two-stage pipelines^37–40^—first detecting entity pairs then classifying relationships as discrete labels—demonstrate critical limitations in handling overlapping triples prevalent in aviation maintenance data. These limitations become evident in statements like “The brake system consists of a brake solenoid valve, a brake controller, and a brake device”, which generate multiple triples sharing identical predicate-subject pairs: < brake solenoid valve, isPartOf, brake system>, < brake controller, isPartOf, brake system>, and < brake device, isPartOf, brake system>. The Cascade Binary Tagging Framework (Casrel)^41,42^ partially addresses this through relationship-as-function modeling but suffers from oversimplified head entity representation, using mere the start and end vectors averages that discard crucial semantic information.

**Fig. 3:**
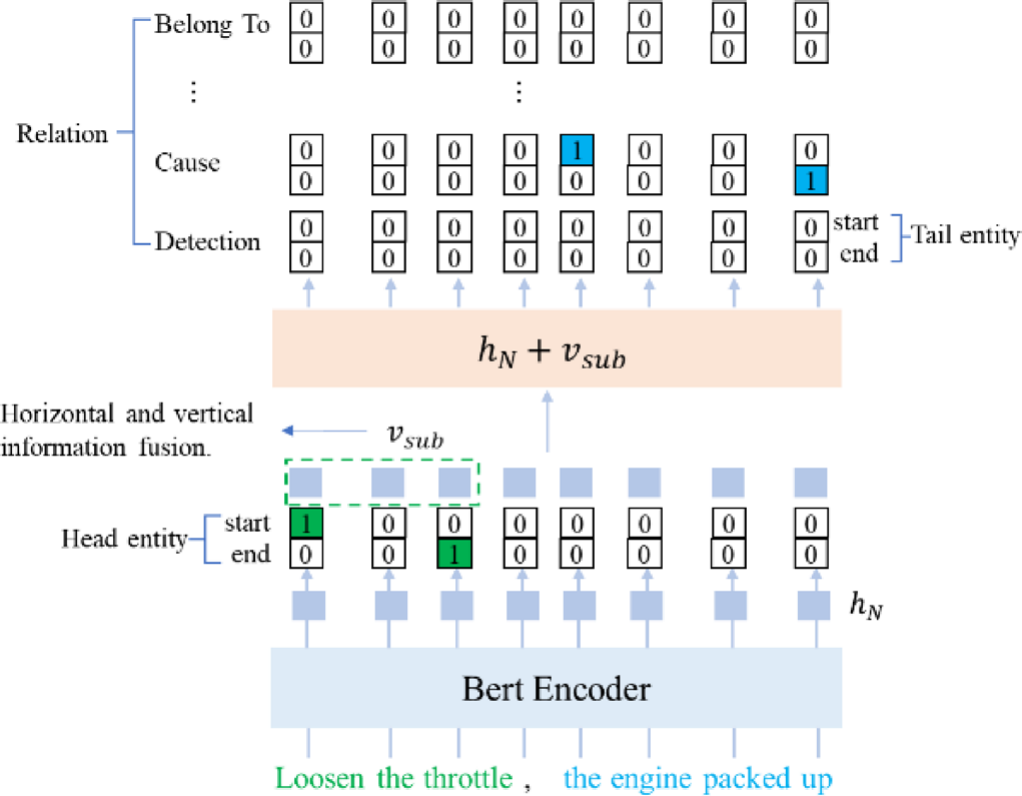
The Hor-Ver-Casrel model architecture. The diagram illustrates the process of extracting a set of < Fault, Cause, Fault > triple instances, where the head entity is “loose the throttle”, the tail entity is “the engine packed up”.

To overcome these limitations, we propose the Hor-Ver-CasRel model—an enhanced Casrel framework integrating horizontal and vertical contextual information for enriched head entity representation. Horizontal context captures intra-sentence entity relationships through syntactic dependencies, while vertical context models inter-sentence semantic patterns across documents. The synergistic fusion of these dimensions elevates head entity representation quality, as visualized in Fig. 3. The algorithm is outlined as follows.

a. Input the text sentences into a pre-trained BERT model, which employs the Transformer architectures as its core encoding mechanism. The model adjusts the weight coefficient matrices based on the inter-character association degree within a sentence to extract word features, that is

1 $$Attention\left( {Q,K,V} \right)=Softmax\left( {\frac{{Q{K^T}}}{{\sqrt {dk} }}} \right)V,$$

where$Q,K,V$represents the character vector matrix, $dk$ represents the embedding dimension, $Attention$denotes the self-attention mechanism function,$Softmax$indicates the activation function, and *T* represents the transpose symbol of the matrix.

b. Based on the vector representation of each character, predict the starting and ending positions of the head entity that

2 $$p_{i}^{{start\_s}}=\sigma \left( {{W_{start}}{F_i}+{b_{start}}} \right)~~$$

3 $$p_{i}^{{end\_s}}=\sigma \left( {{W_{end}}{F_i}+{b_{end}}} \right)$$

where ${F_i}$ represents the vector of the *i *-th character, $p_{i}^{{start\_s}}$ and $p_{i}^{{end\_s}}$ denote the probability of the *i*-th character being the start position of the head entity, respectively. The weight matrices ${W_{start}}$ and ${W_{end}}$, along with their corresponding biases ${b_{start}}$ and ${b_{end}}$, are employed to predict the head entity’s start and end positions, while $\sigma$ represents the fully connected layer responsible for integrating these predictions into the final output.

c. Based on the head entity predicted in the previous step, let ${F_a}$, ${F_b}$ be the word vector at the start and end position of the head entity. Through information fusion in both horizontal and vertical directions, obtain the vector representations for horizontal $v_{{sub}}^{{hor}}$ and vertical $v_{{sub}}^{{ver}}$ directions, respectively. That is

4 $$v_{{sub}}^{{hor}}=average\left( {{F_a},{F_b}} \right)$$

5 $$v_{{sub}}^{{ver}}=\sigma ({W_{sub}}[sum\left( {{F_a}} \right),sum\left( {{F_b}} \right)]+{b_{sub}})$$

Further, concatenate these two vector to obtain the final information representation of the head entity, ${v_{sub}}$. That is 

6 $${v_{sub}}=v_{{sub}}^{{hor}}+v_{{sub}}^{{ver}}$$

d. Construct a mapping function for each relation using the vector representation of each character and the predicted head entity for predicting the tail entity, that is

7 $$p_{i}^{{start\_o}}=\sigma \left( {W_{{start}}^{r}\left( {{F_i}+{v_{sub}}} \right)+b_{{start}}^{r}} \right)$$

8 $$p_{i}^{{end\_o}}=\sigma \left( {W_{{end}}^{r}\left( {{F_i}+{v_{sub}}} \right)+b_{{end}}^{r}} \right)~$$

where $p_{i}^{{start\_o}}$ and $p_{i}^{{end\_o}}$ denote the start and end position labels of the predicted tail entity for the *i*-th character, The weight matrices $W_{{start}}^{r}$ and $W_{{end}}^{r}$, paired with biases $b_{{start}}^{r}$and $b_{{end}}^{r}$, predict these positions under the relation mapping.

The Hor-Ver-Casrel model’s encoding process is completed via maximum likelihood estimation. The likelihood function combines two components that.

9 $$\:L\left(\theta\:,\phi\:r\right)={logP}_{\theta\:}\left(s|X\right)+{logP}_{\phi\:r}\left(o|s,X\right)$$

where $\:{P}_{\theta\:}\left(s|X\right)$ denotes the likelihood function of head entity prediction, $\:{P}_{\phi\:r}\left(o|s,X\right)$ denotes the likelihood function of relation and tail entity prediction. That is

$$\:{P}_{\theta\:}\left(s|X\right)=\prod\:_{t\in\:\left\{start\_s,end\_s\right\}}\sum\:_{i=1}^{L}{\left({p}_{i}^{t}\right)}^{I\left\{{y}_{i}^{t}=1\right\}}{\left(1-{p}_{i}^{t}\right)}^{I\left\{{y}_{i}^{t}=0\right\}}$$

$$\:{P}_{\phi\:r}\left(o|s,X\right)=\prod\:_{t\in\:\left\{start\_o,end\_o\right\}}\sum\:_{i=1}^{L}{\left({p}_{i}^{t}\right)}^{I\left\{{y}_{i}^{t}=1\right\}}{\left(1-{p}_{i}^{t}\right)}^{I\left\{{y}_{i}^{t}=0\right\}}$$

$${{\varvec{\uptheta}}}=\left\{ {{W_{start}},{b_{start}},{W_{end}},{b_{end}}} \right\}, \varphi r=\left\{ {W_{{start}}^{r},b_{{start}}^{r},W_{{end}}^{r},b_{{end}}^{r},{W_{sub}},{b_{sub}}} \right\}$$

Here, $\{ y_{i}^{t}=1\}$ indicates that the i-th character is the start/end position of the head/tail entity, *L* is the sentence length, *I* is the indicator function.

**Fig. 4:**
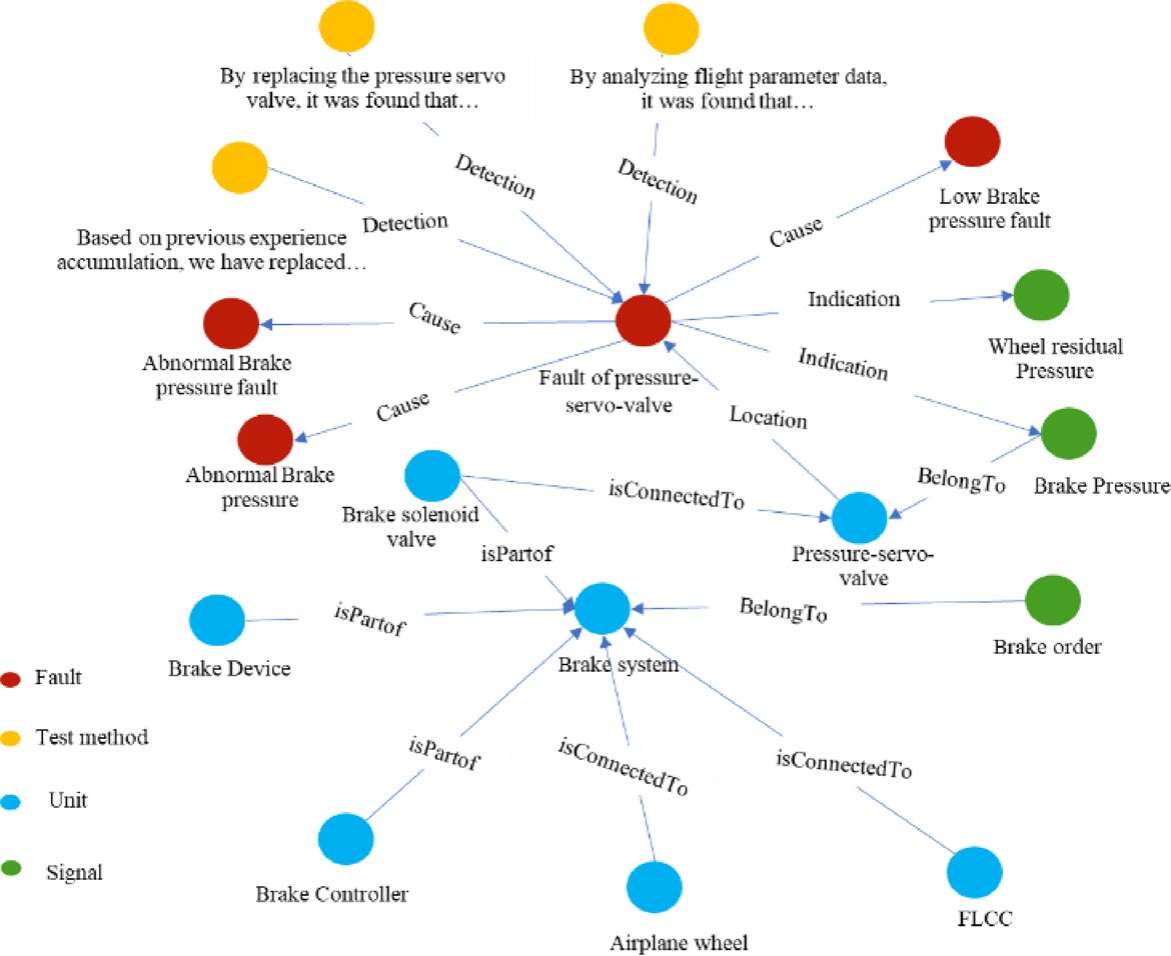
The local fault knowledge graph of a certain type of UAV system, Circles of different colors represent different types of entities, and edges represent different types of relationships, where orange circles denote Test method, red circles denote Fault, blue circles denote Unit, and green circles denote Signal.

Using fault knowledge extraction technology, we construct a fault knowledge graph for a certain type of aircraft. Figure 4. shows the local fault knowledge graph of a certain type of UAV system, which captures critical information such as system architecture, signal parameters, associated faults, and fault detection methods. The constructed graph provides a foundation for subsequent research on LLM-based reasoning over knowledge graphs.

### B. HybirdRAG method based on Multi-Dimensional retrieval

The retrieval stage represents a critical component in RAG-based intelligent troubleshooting systems, where its primary objective is to deliver accurate responses to user queries by effectively extracting relevant information from a well-constructed knowledge base. As retrieval performance directly determines the quality of RAG-generated outputs, our methodology employs a novel multi-dimensional approach that synergistically combines three complementary retrieval techniques. Our framework establishes knowledge graph retrieval as the foundational element, leveraging its rich semantic relationships to capture deep fault characteristics. This core retrieval layer is augmented by vector retrieval and BM25 algorithms, which enable efficient extraction of supplementary information from unstructured data sources. To further optimize retrieval quality, we implement a sophisticated re-ranking mechanism that evaluates and refines initially retrieved contexts, ensuring optimal relevance and precision in the final output. This integrated multi-retrieval strategy can screen out the most decisive information from large-scale knowledge repositories, significantly enhancing the efficiency and reliability of fault troubleshooting.

### 1) Primary retrieval based on KG exploration chain

KG retrieval exploits graph structures to identify the most relevant paths for query resolution. Formally, given a query *q* and domain-specific KG *G*, the retrieval task aims to find the optimal path ${w_z}$ that maximizes the probability of answering *q*:

10 $${\text{IR}}=\mathop {\arg \hbox{max} }\limits_{{{w_z}}} P({w_z}|q,G)$$

Inspired by the graph-based LLM reasoning work^34–36^, we propose a Chain of Exploration with Planner-Agent for fault knowledge graph. Our strategy leverages a planner-agent framework to orchestrate exploration tasks, dynamically adjusting its exploration task based on intermediate results and ensuring context-aware, efficient traversal of the graph, as detailed in Algorithm 1. The algorithm is structured into three primary phases: planning, execution, and self-reflection. In the planning phase—the core component of the algorithm—two key tasks are performed. First, the system evaluates the current state of the query and designs a sequence of exploration tasks $\mathcal{B}$ that are tailored to the structure and content of the knowledge graph. These tasks are organized by a planner-agent utilizing the graph exploration tools $\mathcal{T}$(namely, explore neighbors, explore paths, and explore patterns as detailed in Algorithms 2–4). Second, the planner evaluates the current retrieval state to decide whether to refine the exploration strategy, expand the search scope, or finalize the response via the LLM. Once the planner decides to explore further, the algorithm transitions to the execution phase, where the planned tasks are carried out. Following execution, the system enters the self-reflection phase in which the LLM assesses alignment between retrieved information and the initial query . If the current traversal sufficiently addresses the query requirements, the system proceeds with the next planning cycle; otherwise, it dynamically adjusts the exploration strategy—either by refining existing tasks or by generating additional tasks to address identified knowledge gaps. This iterative process continues until a conclusive answer is reached or the maximum iteration limit is met.

**Figure:**
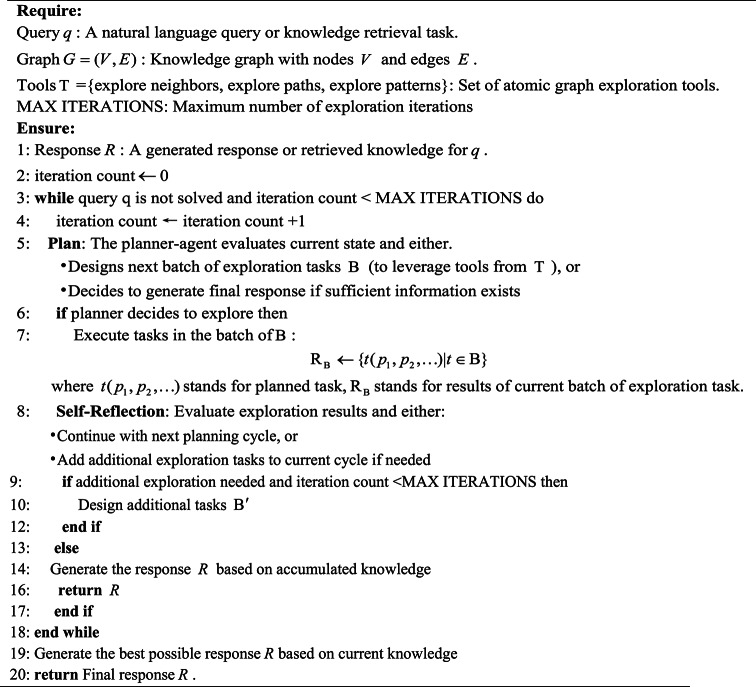
**Algorithm 1 Chain of Exploration with Planner-Agent**

In contrast to conventional GraphRAG approaches that typically operate on schemeless triple graphs^33^, our Chain of Exploration (CoE) framework leverages a structured schema and rich relational semantics within knowledge graph , offering superior performance for complex aircraft fault diagnosis. The innovation of CoE centers on the Zero-Shot Planning paradigm, which utilizes a domain-specific language (DSL) for sophisticated graph reasoning (see Appendix B for DSL details). In this paradigm, we define succinct commands—such as exploring neighbors, paths, or patterns—that can be seamlessly chained together by a large language model. These commands are interpreted by an execution mechanism that translates the DSL into precise graph exploration operations with minimal overhead. This approach not only differentiates CoE from standard GraphRAG solutions but also optimizes its performance for advanced reasoning tasks, making it ideal for handling intricate, real-world challenges. By integrating the Zero-Shot Planning paradigm with DSL, our method eliminates the need for task-specific fine-tuning, enabling rapid adaptation to diverse diagnostic problems while maintaining high efficiency and interpretability.

**Figure:**
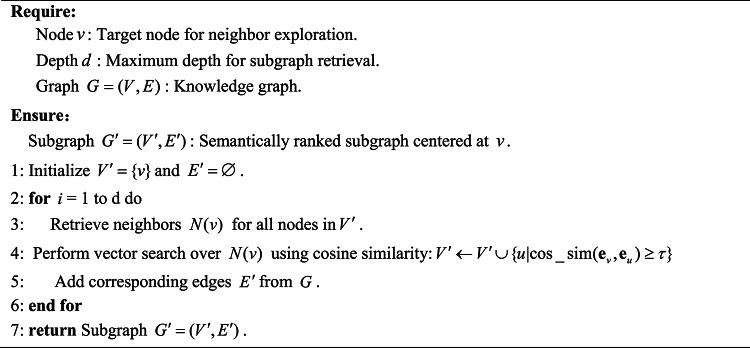
**Algorithm 2 Explore neighbors(node, depth)**

**Figure:**
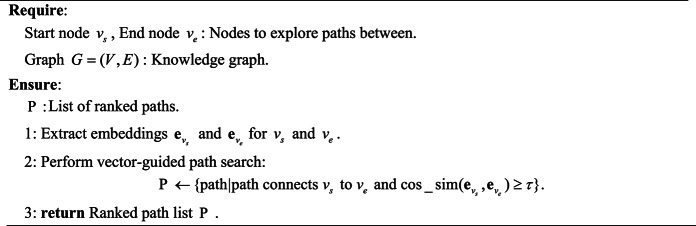
**Algorithm 3 Explore paths(start, end)**

**Figure:**
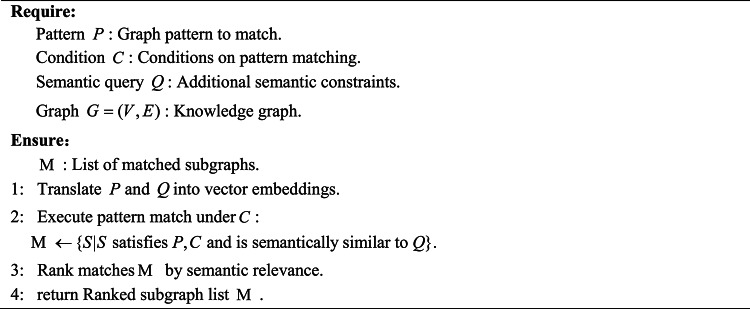
**Algorithm 4 Explore patterns(pattern, condition, query)**

### 2) Complementary retrieval based on vectors and BM25

To fully leverage the original fault descriptions in aircraft technical documentation, we propose a complementary retrieval method based on vector similarity and BM25 algorithms to augment the knowledge obtained through graph retrieval.

#### a. Vector retrieval

**Fig. 5:**
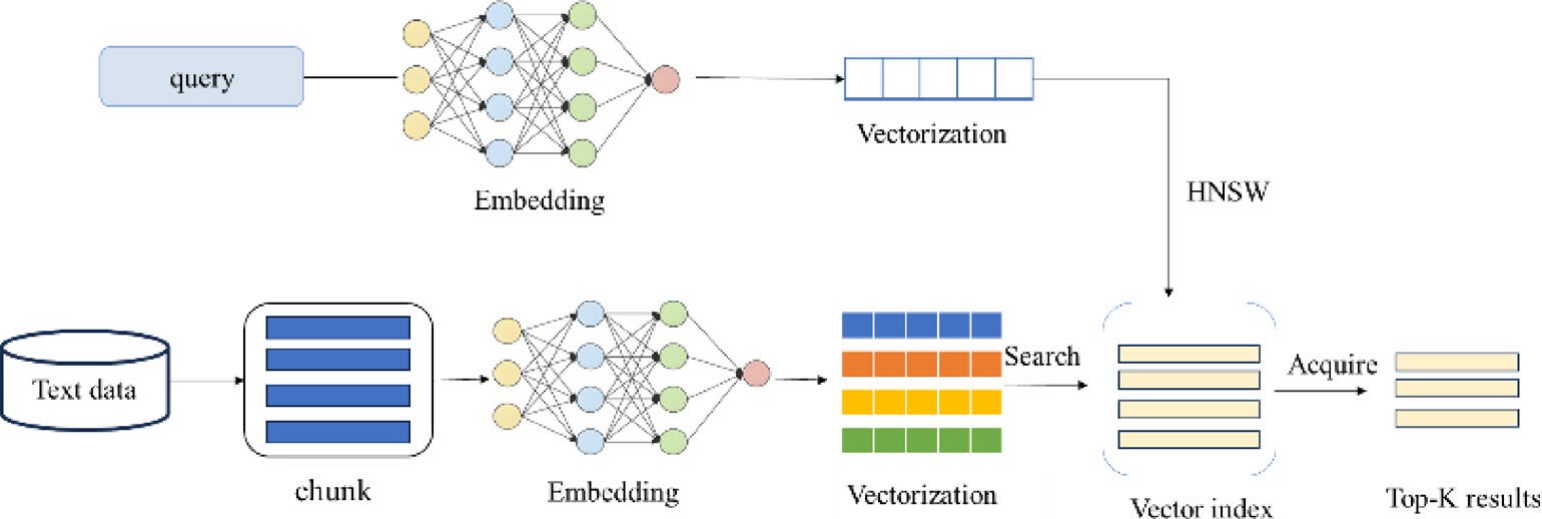
Vector Retrieval Framework Diagram. The framework converts fault text data into vector representations via embedding techniques to construct an index, then retrieves the Top-K most similar results from the vector index for user queries using the HNSW algorithm.

Vector retrieval employs semantic similarity matching, with its workflow depicted in Fig. 5. This method initially processes fault text data through segmentation and chunking, followed by vector representation using advanced embedding techniques^43^. The system then constructs an efficient search index utilizing Hierarchical Navigable Small World (HNSW) graphs^44^, enabling high-performance approximate nearest neighbor search. When processing user queries, the same embedding model generates vector representations that maintain semantic alignment with the indexed content. The HNSW algorithm subsequently retrieves the Top-K most semantically similar text passages, combining the nuanced semantic understanding of embeddings with the computational efficiency of modern approximate nearest neighbor search techniques. This dual advantage makes the approach particularly effective for handling large-scale fault text corpora while ensuring both retrieval speed and result relevance.

#### b. BM25 retrieval

The BM25 algorithm establishes itself as a probabilistic retrieval framework that substantially improves upon traditional TF-IDF methods^45^. At its core, this algorithm evaluates document relevance through a sophisticated interplay of three critical factors including the frequency of query terms within documents, their inverse document frequency across the entire collection, and the length of the document. Within RAG systems, BM25 has proven particularly valuable for enhancing recall performance when employed alongside vector retrieval techniques^46^. For a query *q* composed of terms ${q_1},{q_2}, \ldots ,{q_N}$ and a document *d*, the BM25 ranking function is as follows:

11 $$Score(d,q)=\sum\limits_{{i=1}}^{N} {{\text{IDF}}} ({q_i}) \cdot \frac{{f({q_i},d) \cdot ({k_1}+1)}}{{f({q_i},d)+{k_1}1(1 - b+b\frac{{|d|}}{{avgdl}})}}$$

where $Score(d,q)$ denotes the relevance score between the document *d *and the query *q*, $f({q_i},d)$ is the frequency of the term ${q_i}$ in the document *d*, $|d|$ is the document length, and $avgdl$ represents the average document length across the corpus. The inverse document frequency ${\text{IDF}}({q_i})$ is computed as $\log ({{(N - n({q_i})+0.5)} \mathord{\left/ {\vphantom {{(N - n({q_i})+0.5)} {(n({q_i})+0.5}}} \right. \kern-0pt} {(n({q_i})+0.5}})+1)$, where *N* is the total number of documents in the collection and $n({q_i})$ is the number of documents containing ${q_i}$. The free parameters ${k_1}$ (typically set between 1.2 and 2.0) and *b *(usually 0.75) control term frequency saturation and document length normalization, respectively.

## 3) Cross-Encoding Re-Ranking

To enhance the relevance and accuracy of retrieved results, we introduce a cross-encoding re-ranking^47^ mechanism that operates on the initial retrieval outputs from heterogeneous sources including knowledge graphs, vector embeddings, and BM25 algorithms. This module serves as a critical refinement stage in our Retrieval-Augmented Generation (RAG) pipeline, effectively filtering irrelevant content and optimizing the ranking of retrieved passages based on their semantic relevance to the input query^48,49^.

**Fig. 6:**
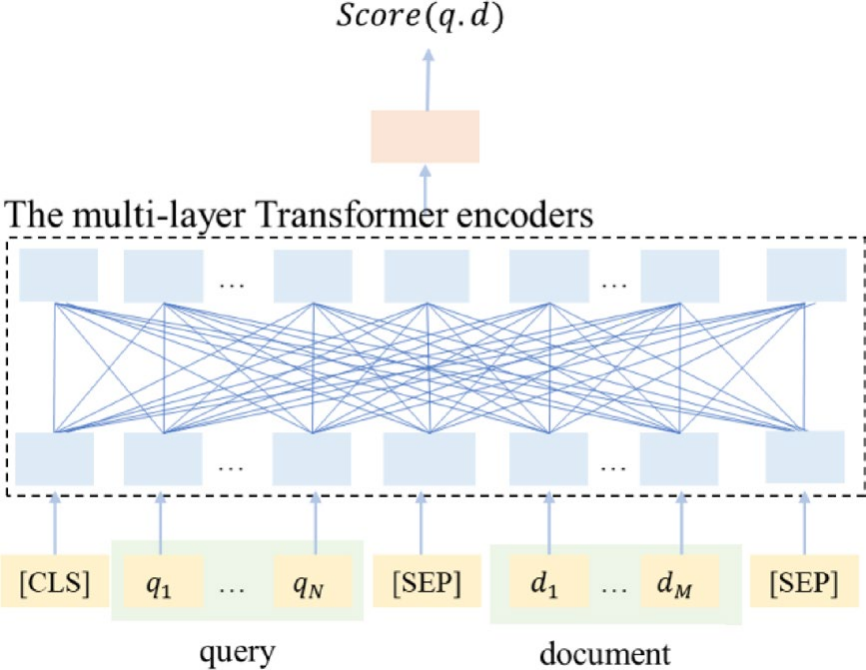
Cross-encoding Re-ranking Model Architecture. The framework processes concatenated query-document sequences (yellow boxes) through multi-layer Transformer encoders (dashed box). The encoded sequence representations (blue box) are used to extract the [CLS] token’s hidden state (orange box), which serves as the joint query-document representation for relevance scoring.

The architecture of the cross-encoder, depicted in Fig. 6, implements a sophisticated relevance scoring system through sequential processing stages. Formally, given a query *q *and document chunk *d*, the model constructs an input sequence following the standard Transformer format:

12 $$Input=[CLS]q[SEP]d[SEP]$$

where [CLS] denotes the classification token and [SEP] represents sequence separators. This composite sequence undergoes deep contextual encoding through multiple Transformer layers:

13 $$H=Transformer\left( {Input} \right)$$

yielding a sequence of hidden states *H*. The model subsequently extracts the hidden state corresponding to the [CLS] token:

14 $${h_{{\text{CLS}}}}=H[{\text{CLS}}]$$

which encapsulates the joint query-document representation. Finally, a feedforward neural network (FFN) processes this representation to generate the relevance score:

15 $$Score\left( {q,d} \right)=FFN( {h_{CLS}})$$

Based on these computed scores, the system returns the top-K most relevant contents for subsequent answer generation.

### C. Answer generation

The LLM synthesizes coherent and contextually relevant responses by processing the top-K re-ranked retrieval results from previous stages. This input concentration mechanism ensures the model receives the most pertinent information for each query. Through carefully designed prompt engineering, the retrieved content is transformed into structured input parameters that enhance the LLM’s contextual comprehension. The model then leverages its language generation capabilities to produce informative, query-specific responses. This generation process is formally expressed as

16 $$a={\text{LM}}(q,Q)$$

where *Q* represents the retrieved information. To optimize response quality, the LLM maximizes the probability of generating the most appropriate answer *a* given the query *q* and the retrieved context *Q*, that is

17 $$\arg {\hbox{max} _{a \in A}}P(a|q,Q)=\prod\limits_{{j=1}}^{m} P ({a_j}|{a_{<j}},q,Q)$$

where *A* denotes the set of all possible answers, *m *is the answer length, and $P({a_j}|{a_{<j}},q,Q)$ represents the conditional probability of each word ${a_j}$ in output sequence given the preceding context ${a_{<j}}$, query *q*, and retrieved information *Q*.

**Fig. 7:**
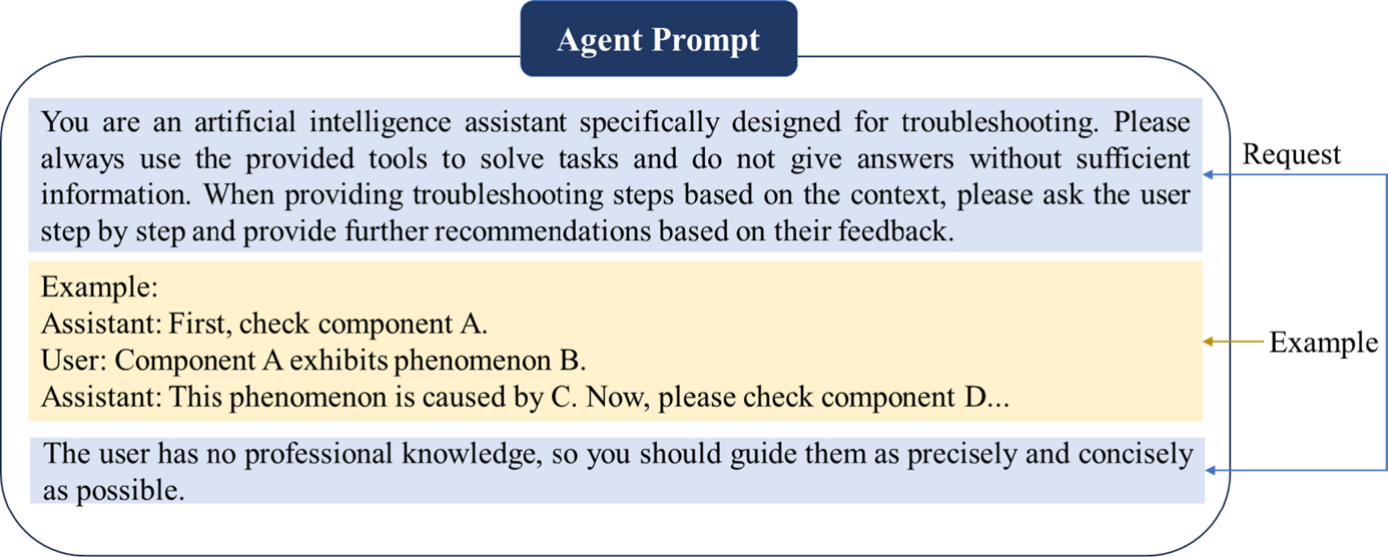
Structured Instruction Prompts for Agent-Guided Troubleshooting. The blue highlighted block represents the request, which specifies the assistant’s mandatory inference rules including step-by-step verification requirements. The yellow highlighted block provides the example, demonstrating the expected interaction sequence. This two-component design ensures context-aware guidance while maintaining strict diagnostic protocols.

To operationalize interactive fault diagnosis, we developed an intelligent troubleshooting assistant within our guided framework. As illustrated in Fig. 7, the assistant implements structured system instruction prompts consisting of explicit request specifications for AI inference and formatted demonstration examples. These prompts enforce a strict step-by-step diagnostic protocol where the assistant must first request user verification of a specified component, then provide subsequent guidance based on the observed phenomenon, and finally proceed to the next logical check point. The design specifically accommodates non-expert users through constrained interaction patterns that prevent unsupervised diagnostic actions.

### Experiment studies

This section evaluates the troubleshooting guidance efficiency of the proposed method in aircraft fault diagnosis scenarios, with experimental dataset derived from the UAV fault knowledge graphs constructed in Method section. The implementation details and results analysis are presented in the following subsections.

### A. Experimental settings

The experiments were conducted on a local machine equipped with four NVIDIA GeForce RTX 3090 GPUs running Ubuntu 22.04 with a 32-core and 256GiB RAM. The large language model (LLM) employed was qwen2-72b^50^. We utilized the constructed UAV fault knowledge graph corpus—consisting of 1,000 fault handling records/articles and 20 MB of fault-related technical text manuals—as the RAG experiment dataset. This study investigates three RAG frameworks for assisted diagnosis: HybridRAG, VectorRAG, and GraphRAG_sub (subgraph retrieval-based). For each query, HybridRAG was configured with a maximum of 5 iterations, returning the most relevant results through top-10 ranking^51^ while maintaining a default subgraph depth of 3. The subgraph depth of 3 was determined through a comprehensive analysis of domain-specific schema design and instance data characteristics, ensuring sufficient knowledge coverage for fault diagnosis queries. When the default retrieval results are insufficient, the planner-agent autonomously adjusts the subgraph retrieval depth to meet task-specific requirements.

In our experiments, we evaluated the methods using four key metrics—F1-score, hallucination rate, end-to-end latency, and RAM utilization—to assess both accuracy and practical deployment feasibility. Specifically, the F1-score quantifies diagnostic accuracy, while the hallucination rate measures the frequency of irrelevant or incorrect outputs generated during inference. End-to-end latency and RAM utilization were used to assess computational efficiency and resource consumption. Notably, traditional statistical significance tests (e.g., confidence intervals, p-values) were not applied to evaluate the robustness of these metrics due to the non-independent and identically distributed (non-IID) nature of textual data, which violates fundamental statistical assumptions. Our metric selection follows established practices in LLM, knowledge graph, and RAG research communities, where absolute performance improvements typically outweigh statistical validation^22,36^.

#### 1) F1-score

The F1-score evaluates answer prediction quality by combining precision and recall metrics. For each diagnostic query, this is formally computed as

18 $${\text{F1(\% )}}=\frac{{2 \times {\text{Precision}} \times {\text{Recall}}}}{{{\text{Precision}}+{\text{Recall}}}} \times 100\%$$

19 $${\text{Precision}}=\frac{{count\_pred}}{{len\left( {\hat {a}} \right)}}$$

20 $${\kern 1pt} {\text{Recall}}=\frac{{count\_pred}}{{len\left( a \right)}}$$

where $\hat {a}$denotes the predicted answer, *a* represents the ground truth answer, $len\left( {\hat {a}} \right)$ and $len\left( a \right)$ indicate the length of the predicted and true answers respectively, and$count\_pred$corresponds to the number of matching tokens between $\hat {a}$ and *a*.

#### 2) Hallucination Rate

The hallucination rate (HR) quantifies the frequency of generating content that does not contain real information. Formally, HR is computed as

21 $${\text{HR(\% )}}=\frac{{\sum\limits_{{i=1}}^{N} {h({{\hat {a}}_i},{a_i})} }}{N} \times 100\% ,{\kern 1pt} {\kern 1pt} {\kern 1pt} {\kern 1pt} {\kern 1pt} {\kern 1pt} i=1,2,\cdots,N$$

where${\hat {a}_i}$ and ${a_i}$ denotes respectively predicted and ground truth answers for the i-th question, *N* denotes the total number of questions, $h({\hat {a}_i},{a_i})$denotes the hallucination score for the i-th question, which is ranged from 0 to 1 and implemented via “LLM as a judge” between ground truth and generated answers by the system.

## *B. Experimental Results*

### 1) Overall performance

The performance of the proposed HybridRAG method and baseline approaches (VectorRAG and GraphRAG_sub) was evaluated using 100 domain-specific fault diagnosis queries derived from real-world aviation scenarios. Experimental results, summarized in Table 1, demonstrate that HybridRAG significantly outperforms its counterparts in both diagnostic accuracy and reliability. Notably, HybridRAG achieves an F1-score of 84.83%, representing a 4% relative improvement over the next-best GraphRAG_sub (80.12%), while reducing the hallucination rate (HR) to 16.67% —a 7% reduction compared to VectorRAG’s 24.05% HR. These results underscore the efficacy of HybridRAG’s hybrid retrieval strategy in minimizing diagnostic errors and improving decision-making precision. Regarding resource consumption, HybridRAG incurs higher computational costs due to the Graph Index system overhead—specifically, a 41.19-second inference latency (versus 32.81–33.51 s for baselines) and 26.12 GiB RAM usage (versus 16.91–18.11 GiB). However, these costs are justified by the substantial accuracy gains. Furthermore, in practical troubleshooting scenarios, the 41.19-second latency per query is acceptable when contrasted with the total troubleshooting duration, which typically requires one hour or more of technician time. Critically, in this domain, diagnostic precision outweighs response speed, as even minor inaccuracies, such as hallucinations that mislead engineers, can derail diagnostic efforts and substantially extend troubleshooting timelines.

**Table 1:** The performance results of some methods.

method	F1-score	HR	Latency^*^	RAM
VectorRAG	76.59%	35.43%	32.81 s	16.91 GiB
GraphRAG_sub	80.12%	24.05%	33.51 s	18.11 GiB
HybirdRAG(ours)	84.83%	16.67%	41.19 s	26.12 GiB

^*^Latency denotes the average time taken to process 100 questions, measured from the start of retrieval to the completion of answer generation.

**Table 2:** The performance results of some methods for new fault.

method	F1-score	HR
VectorRAG	69.54%	46.05%
GraphRAG_sub	75.69%	23.76%
HybirdRAG(ours)	81.50%	15.01%

In addition, we discovered through BM25 retrieval analysis that 82 out of 100 collected fault queries were entirely novel—their corresponding fault cases, root causes, or solutions were absent in the training corpus, but their underlying operating principles and structural configurations were fully covered in the dataset. We thus designated these 82 query pairs as a dedicated benchmark for assessing generalization, with results presented in Table 2. The findings demonstrate that graph-based reasoning outperforms baseline methods for unseen queries. Specifically, HybridRAG exhibits a smaller performance decline compared to historical data in terms of F1-score (84.83% → 81.50%) and HR (16.67%→ 15.01%), suggesting a more effective extraction of the contextual information required for robust knowledge reasoning in unseen fault scenarios.

### 2) Robustness

**Fig. 8:**
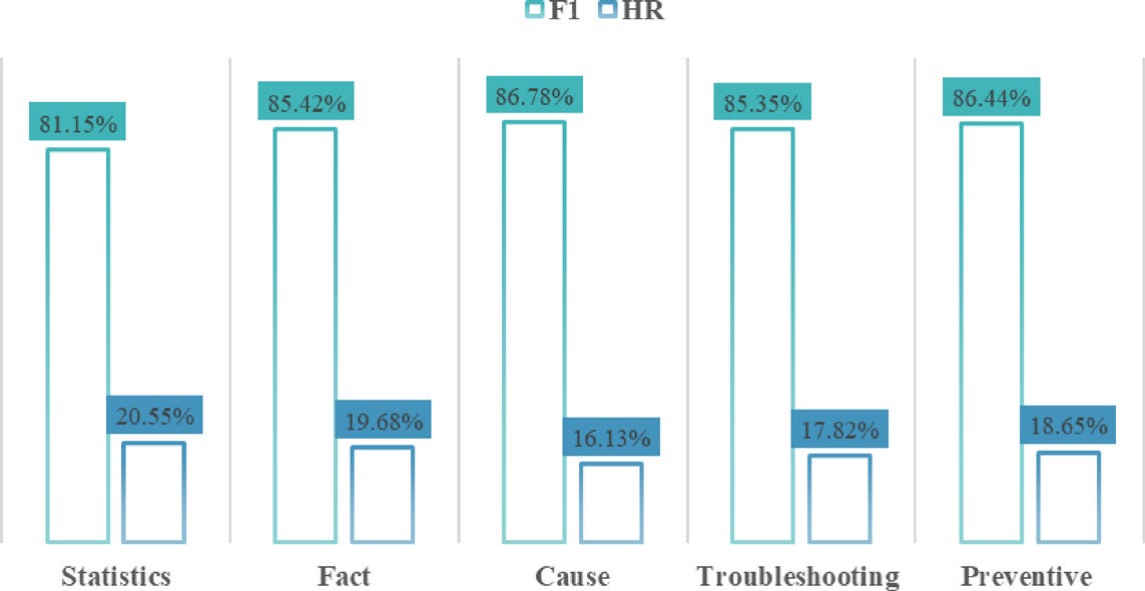
The performances of five categories of fault-related questions on HybirdRAG method.

To validate the robustness of the HybirdRAG method, we collected five categories of aircraft fault-related questions as detailed in Table 3, with 100 distinct test queries per category. Figure 8 presents the evaluation results, showing that the proposed method consistently achieves F1-scores above 80% while keeping hallucination rates below 30% across all question categories, which confirms its stable diagnostic performance. However, HybridRAG exhibits performance variations across different question types due to varying inference complexities. Statistical questions, in particular, are more challenging due to their complex reasoning requirements. For instance, when answering *“What percentage of a specific root cause’s frequency accounts for all possible causes of a given fault phenomenon?“*, the model must first infer all plausible root causes and then calculate their relative frequencies. This two-step process is prone to hallucinations, such as inventing non-existent cause distributions or miscounting statistical patterns.

**Table 3:** The question categories and examples.

Category	Example
Statistics	How many cases have occurred where the left brake pressure is too low?
Fact	What are the components of the brake system?
Cause	What causes the left normal brake pressure to be too low?
Troubleshooting	How to troubleshoot the left brake pressure being too low?
Preventive	How to maintain normal brake pressure on the left side?

Further analysis reveals that HybridRAG exhibits performance limitations in specific fault scenarios, particularly for root cause identification and judgment of abnormal signal parameter characteristics. These challenges originate from both knowledge graph schema ambiguities and corpus data quality issues. The schema design introduces reasoning inconsistencies when edge types are misinterpreted—the BelongTo relationship between Signal and Unit entities is occasionally applied incorrectly to Unit-Unit connections, while the explicitly defined Cause relationship between Fault entities is sometimes misconstrued as linking Fault to Fault Case, despite prompt-based constraints. Parallel to these schema challenges, the original corpus contains unstructured maintenance records featuring vague descriptions, inconsistent terminology, and missing technical specifications. These data quality limitations introduce substantial noise during information retrieval, particularly affecting experience-based articles written by maintenance personnel.

The experimental results conclusively demonstrate that HybridRAG’s innovative integration of multi-dimensional retrieval strategies yields significant improvements in fault diagnosis efficiency. This enhanced performance, particularly evident in complex diagnostic scenarios, underscores the methodological superiority of the hybrid approach and its critical value for practical aviation maintenance applications. Although schema ambiguities and data quality limitations present identifiable challenges, they represent addressable constraints rather than fundamental flaws—offering clear pathways for future methodological refinement. Collectively, these empirical results establish HybridRAG as a practically viable solution, providing a robust foundation for its ongoing implementation in aircraft fault diagnosis systems.

#### C. Agent-Guided troubleshooting

**Fig. 9:**
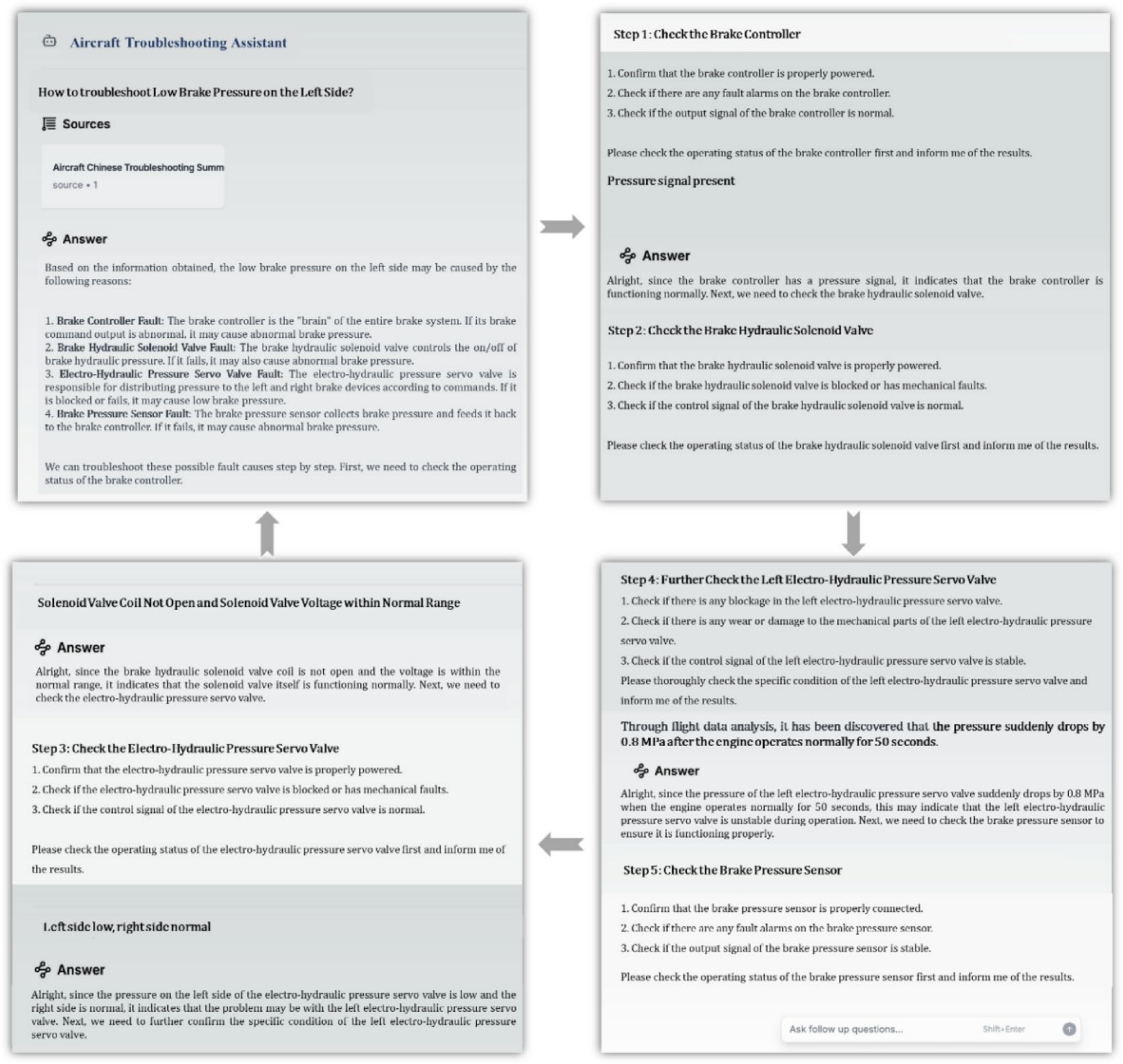
Interactive fault diagnosis process guided by the HybridRAG agent.

Based on the proposed HybirdRAG method and a designed system prompt framework, we developed an interactive troubleshooting guidance agent. As demonstrated in Fig. 9, the agent guides users through a structured fault diagnosis process. For example, when a user inquiries about “how to troubleshoot low pressure in the left normal brake system,” the agent provides all possible causes of the fault along with detailed troubleshooting steps. The user follows these steps to perform tests and reports the results back to the agent, which then determines and suggests the next steps until the specific fault cause and component are identified. This guided fault diagnosis agent emulates an expert’s thought process. Compared to traditional question-and-answer systems, it more effectively guides users through complex fault diagnosis tasks. This interactive model both troubleshooting efficiency and user experience, demonstrating the practical value of this technology in aircraft fault diagnosis applications.

## Conclusion

In this paper, we propose a guided fault diagnosis method for aircraft based on the HybridRAG framework, which integrates knowledge graphs and large language models. By combining multiple retrieval strategies, including VectorRAG, BM25, and graph-based reasoning, our method enhances inference capabilities for complex fault diagnosis through the synergistic use of structured knowledge and unstructured data. We further develop an intelligent diagnostic agent that implements a multi-turn conversational functionality to guide maintenance personnel through troubleshooting process, significantly improving interactivity, and operational efficiency in real-world aviation maintenance scenarios.

However, despite its effectiveness, our experiments reveal that the proposed method can still fail due to issues such as schema ambiguities and low data quality. To address these, we plan to refine the domain-specific schema by leveraging expert knowledge as a foundation and using large language models (LLMs) to identify inconsistencies and gaps in the current design, thereby eliminating ambiguities and ensuring completeness—a one-time upfront cost since the finalized schema remains unchanged during actual deployment. Furthermore, we will incorporate reinforcement learning (RL) techniques to train the model to critically evaluate the reliability of input data, adopting an offline-training/online-inference framework that ensures only marginal inference costs during deployment post-training, enabling it to prioritize credible data sources during the reasoning process and reduce the impact of noisy inputs. These improvements will enhance HybridRAG’s robustness without compromising deployability.

Beyond addressing the current limitations, we recognize that the scope of diagnostic data needs extension. While this study focuses on unstructured text data, real-world aircraft maintenance relies on diverse data sources including fault images, diagnostic videos, and sensor readings. Integrating these modalities will enable more comprehensive fault analysis—particularly for complex cases where visual inspection or sensor trends provide critical supplementary evidence. Moving forward, we will explore unified multimodal reasoning methods to jointly interpret textual, visual, and time-series data. This expansion is expected to improve robustness in handling ambiguous or rare faults while broadening the system’s applicability across different maintenance scenarios.

## Electronic supplementary material

Below is the link to the electronic supplementary material.

Supplementary Material 1

## Data Availability

The datasets analysed during the current study are not publicly available due to confidentiality and sensitive information but some de-identified data can be available partly from the corresponding author on reasonable request.
