# Supplementary material for: An intelligent guided troubleshooting method for aircraft based on HybirdRAG
Source: Sci Rep. 2025 May 22;15:17752. doi: 10.1038/s41598-025-02643-2 (PMC12098894; doi:10.1038/s41598-025-02643-2)
Supplement: Supplementary file 1 — Supplementary Material 1 [file 41598_2025_2643_MOESM1_ESM.docx]

# APPENDIX A

A detailed explanation of entities, relationships, and attribute in graph schema, as shown in Table AI-AIII.

TABLE AI Types and definitions of entity

| **Entity** | **Definition** | **Attributes** |
| --- | --- | --- |
| Unit | A specific entity element at a particular level of the system (e.g., subsystem, component, part, etc.). A unit can be composed of several subunits or can itself be a subunit of a larger unit. | Includes attributes such as model, manufacturer, image, etc. |
| Signal | A measurable parameter that can indicate many possible faults through signal anomalies. | Includes attributes such as standard value, upper limit, lower limit, etc. |
| Fault | A description of any abnormal event, such as functional failure, parameter drift, mechanical damage, etc. It includes aspects like fault manifestation, fault mode, and fault cause. | / |
| Fault case | A specific real-world instance of a fault event. A fault event may occur multiple times at different times and locations, each occurrence being a separate fault case. | Includes multiple attributes such as fault time, fault location, fault impact, etc. |
| Test method | A method capable of detecting a specific fault event, serving as key knowledge to guide maintenance engineers in quickly locating the faulty unit. | Includes two attributes that operation and judgment. |

TABLE AII Types and definitions of attributes

| **Attribute Type** | **Belongs to Entity** | **Definition** | **Example** |
| --- | --- | --- | --- |
| Upper Limit | Signal | The upper limit of a characterizing signal within the standard range | 2.2 Mpa (Lubricating Oil Pressure) |
| Standard Value |  | The standard value of a characterizing signal | 2 Mpa (Lubricating Oil Pressure) |
| Lower Limit |  | The lower limit of a characterizing signal within the standard range | 1.8 Mpa (Lubricating Oil Pressure) |
| Image | Unit | The image of the component unit | Structural diagram of the component unit, etc. |
| Id |  | The model type of the component unit | HY-123 |
| Manufacturer |  | The production or overhaul facility of the component unit | Manufacturer XY |
| Operation | Test method | The specific procedure of the inspection method | Use an instrument to check lubricating oil pressure |
| Judgment |  | Determine if the inspection results are normal | Lubricating oil pressure is above the standard range |
| Fault Cause | Fault Case | The ultimate cause of the fault occurrence | Pressure sensor failure |
| Fault Time |  | The date of the fault occurrence | May 13, 2022 |
| Fault Location |  | The location and environment where the fault occurred | Airport XY |
| Fault Timing |  | The timing of the fault occurrence | During flight, upon landing |
| Fault Impact |  | Whether the fault occurrence affects mission or safety | Incorrect flight, affects mission, no impact |
| Fault Unit |  | The field replaceable unit where the fault occurred | Metal chip sensor, lubricating oil tank, thrust nozzle flap, thermocouple |
| Specialty |  | The specialty corresponding to the fault | Mechanical, special equipment, avionics |
| Maintenance Measure |  | The repair method taken to restore the engine to a healthy state after the fault | Return to factory for repair, replace parts, polish, swap components |

TABLE AIII Types and definitions of relation

| **Relationship** | **Definition** | **Head Entity** | **Tail Entity** |
| --- | --- | --- | --- |
| Cause | Represents the causal relationship between faults | Fault | Fault |
| Detection | A certain fault event can be detected by a specific inspection method | Fault | Test method |
| IsPartof | A certain component unit is part of another component unit | Unit | Unit |
| isConnectedTo | A physical contact or signal input-output relationship between a component unit and another unit | Unit | Unit |
| BelongTo | A certain signal parameter belongs to a specific component unit | Signal | Unit |
| Location | A certain fault occurs at a specific component unit | Fault | Unit |
| Indication | A certain fault can be characterized by a specific signal | Fault | Signal |
| Relevance | A fault case is associated with a specific fault event | Fault Case | Fault |

# APPENDIX B

| This DSL implements a hybrid approach to graph querying, combining semantic-based natural language processing with boolean filter conditions. It supports three fundamental operations:  1. Neighborhood Exploration (Get Neighbours)  2. Path Finding (FIND PATH)  3. Pattern Matching  Key features:  - Dual-mode queries: Combines natural language semantic queries with precise boolean filters  - Flexible filtering: Supports multiple operators (eq, gt, lt, gte, lte, in, contains)  - Node-specific filtering: Allows detailed constraints for pattern matching  下面是实现的细节  """  You are an expert graph database analyst. Your task is to plan up to {{max_steps}} steps of graph exploratior.  Available Actions:  1.Get Neighbours: Retrieve adjacent nodes and relationships for specified entities.  2.FIND PATH: Discover connections between two entities in the graph.  3.Pattern Matching: Execute a custom pattern matching query when the above actions are insufficient.  Key Guidelines:  1.Use the provided node types and edge types from the schema in your plan.  2.For Pattern Matching:  -Use ONLY the actual node types from the schema in the pattern.  -Use the node queries section to specify semantic search criteria for specific nodes in the pattern  3.Prefer 'Get Neighbours' and 'FIND PATH' for entity-centric questions.  4.Use 'Pattern Matching' only when the other actions cannot adequately express the required information.  5.Ensure each step directly contributes to answering the question.  Output Format (YAML):  ```yaml  - name: [Step description]  actions:  - name: [Action type]  [Action-specific parameters]  filter_conditions: # Optional new section for boolean filters  type: [Entity type]  properties:  [property_name]:  operator: [eq\|gt\|lt\|gte\|lte\|in\|contains]  value: [property_value]  ```  Example:  ```yaml  # Example 1: Get Neighbours - Finding abnormal voltage output by the DC power supply, with the normal voltage range being 26~30V.  - name: Find Abnormal voltage output by the DC power supply.  actions:  - name: Get Neighbours  entities:  - " DC power supply"  semantic_query: " Abnormal voltage output by the DC power supply "  filter_conditions:  type: Signal  properties:  priority:  operator: gt  value: 30  operator: lt  value: 26  # Example 2: Path finding - the causal path between insufficient hydraulic pressure and landing gear retraction failure.  - name: Find the causal path between two faults  actions:  - name: FIND PATH  start: " insufficient hydraulic pressure"  to: " landing gear retraction failure"  # Example 3: Pattern matching - Test method of Landing gear fault  - name: Test method of unit fault  actions:  - name: Pattern Matching  pattern: (t:Unit)<-[:Location]-(m:Fault)<-[:detection]-(c: Test method)  semantic_query: " What test methods are used to detect faults in the landing gear "  """ |
| --- |
